# Impact of Agro-industrial Byproducts on Bioconversion, Chemical Composition, in vitro Digestibility, and Microbiota of the Black Soldier Fly (Diptera: Stratiomyidae) Larvae

**DOI:** 10.1093/jisesa/ieaa148

**Published:** 2021-01-22

**Authors:** Gianluca Galassi, Costanza Jucker, Pietro Parma, Daniela Lupi, Gianni Matteo Crovetto, Sara Savoldelli, Stefania Colombini

**Affiliations:** 1 Department of Agricultural and Environmental Sciences—Production, Landscape, Agroenergy (DiSAA); 2 Department of Food, Environmental and Nutritional Sciences (DeFENS), University of Milan, Milan, Italy

**Keywords:** *Hermetia illucens*, fatty acids composition, microbiota, nutritive value, larval biomass

## Abstract

The interest in using byproducts from agro-food industries as a rearing substrate for insects is increasing rapidly. We investigated the influence of byproducts of vegetal origin (okara—a byproduct of soy milk production, maize distillers with solubles, brewer’s grains), used as rearing diet for black soldier fly larvae (BSFL), on the following parameters: biomass production, substrate reduction (SR), nutritional profile and in vitro digestibility, and larval gut microbiota. Hen diet was used as a control substrate. The highest larval biomass was collected on maize distillers, whereas the highest SR was observed on okara. The rearing substrate affected ash, ether extract, and chitin larval content. The BSFL reared on okara were characterized by a lower lauric acid content (17.6% of total fatty acids). Diets also influenced in vitro crude protein digestibility (%) for monogastrics, with the highest values for BSFL reared on maize distillers (87.8), intermediate for brewer’s grains and okara BSFL, and the lowest for hen BSFL (82.7). The nutritive value for ruminants showed a lower Net Energy for lactation for BSFL reared on hen diet than okara and dried maize distillers BSFL. The different byproducts showed an influence on the larval gut microbiota, with a major bacterial complexity observed on larvae fed with the hen diet. The neutral detergent fiber concentration of dietary substrate was negatively correlated with Firmicutes and Actinobacteria relative abundance. Insects valorized byproducts converting them into high-value larval biomass to be used for feed production. The results evidenced the effects of the tested byproducts on the measured parameters, underling the chemical composition importance on the final insect meal quality.

Growing human population and rising living standards are leading to an increase in food production, particularly of animal origin products that will dramatically increase up to 70% (Oonincx and de Boer 2012, FAO 2017). The exploitation of natural resources has led to high environmental impacts, competition in the food-feed production, and increased livestock farming costs. Insects are considered a new high-quality protein source that can contribute to global food security (Rumpold and Schlüter 2013, Pinotti et al. 2019). Many insect species showed a high content of proteins, lipids, energy, and micronutrients and can replace (partially or totally) soybean meal in animal feeding, particularly in aquaculture production (Barragan-Fonseca et al. 2017, Gasco et al. 2020). One of the main advantages of insect feed production is that insects can be fed with a wide range of byproducts from the agro-food industry, whose elimination has an economic and environmental cost (Bava et al. 2019). Due to their ability to convert low-value organic substrates into macromolecules of high nutritional value, insects represent an excellent alternative source to manage and valorize waste and byproducts, sustaining a circular economy (Cappellozza et al. 2019, Fowles and Nansen 2020, Jucker et al. 2020). Seven insect species (two flies, two mealworms, and three cricket species) have been authorized as fish feed by EU commission regulation (2017/893-24/05/2017). Moreover, insects as a feed ingredient for poultry diets are now being explored for research studies (Schiavone et al. 2017), whereas DiGiacomo and Leury (2019) evidenced that insects pose an opportunity to develop a new sustainable feed source for pig producers in Australia. The use of insect meals for ruminants is currently not allowed in European or North-American countries; however, in several Asian countries, insects are historically considered food and feed and used as a protein source. Few studies investigated the in vitro rumen digestibility of insect larvae as feed for ruminants (Makkar et al. 2014, Jayanegara et al. 2017, Campbell et al. 2020).

The fly *Hermetia illucens* L. (Diptera: Stratiomyidae), commonly known as the black soldier fly (BSF), is one of the most promising species as feed for livestock (Barragan-Fonseca et al. 2017, Gasco et al. 2020). Larvae can grow on various substrates characterized by wide variability in chemical composition, such as fruit and vegetable wastes, kitchen wastes, and brewery’s byproducts (Jucker et al. 2017, 2019; Nyakeri et al. 2017; Varelas 2019). The nutritional composition of the rearing substrate influences the growth performance of black soldier fly larvae (BSFL) both in terms of growth rate and survival of the different preimmaginal instar. Adults seem to be less influenced by feed quality as their survival mainly depends on the resources accumulated by larvae (Lupi et al. 2019). Usually, low-protein and low-energy diets promote a slower larval development, whereas optimal growth is observed with a more balanced diet. The chemical composition of the growing substrate also influences the quality of the final BSFL in terms of chemical composition. Makkar et al. (2014) reported a wide range of variation (% DM) from 15.0 to 34.8 for ether extract (EE) and from 14.6 to 28.4 for ash for BSFL. The review by Barragan-Fonseca et al. (2017) reports a protein content (% DM) from 38.5 to 62.7, whereas the crude fat is between 6.63 and 39.2.

Rearing substrate also significantly impacts the microbial gut community (Jeon et al. 2011, Bruno et al. 2019). Microbiota composition impacts insect health and performance and has to be considered to optimize biomass yield (De Smet et al. 2018). However, there is still a paucity of information on BSF intestinal microbiota, and only a few studies were conducted to investigate the relationship between rearing substrate and BSF gut microbiome (Jeon et al. 2011, Bruno et al. 2019, Jiang et al. 2019, Callegari et al. 2020).

In a previous study, Bava et al. (2019) verified the suitability of byproducts collected from the local market-okara (a byproduct of soy milk production), dried distillers grains with solubles (maize distillers), and brewer’s grain with trub- as growing substrates for BSFL, assessing the larval survival, final larval weight, and bio-reduction ability. Objectives of the present study were to 1) analyze the influence of the same substrates tested by Bava et al. (2019) on larval development time and the larval biomass produced; 2) assess the nutritional composition of the larvae, with particular attention to the fatty acid composition; 3) evaluate in vitro digestibility of larval meal for monogastrics and ruminants; and 4) investigate the impact of the rearing substrate on the larval microbiota.

## Materials and Methods

### Insect Rearing and Experimental Trials

BSFL used in the experimental trials were provided from a laboratory colony located at the University of Milan (Jucker et al. 2017). Neonate larvae were fed on hen feed for 2 d before being transferred with a fine brush on the different experimental growing substrates.

The following substrates were supplied as feeding for the larvae: okara (a byproduct of soy milk production; Triballat Italia, Fidenza, PR, Italy), dried distillers grains with solubles (maize distillers; Brioni SpA, Castenedolo, BS, Italy), and brewer’s grains (Birrificio Lambrate, Milan, Italy). Brewer’s grains were a mixture of brewer’s grains and trub (another byproduct from the brewing industry) in a 1:1 proportion (Bava et al. 2019). Okara and brewer’s grains were frozen and positioned at room temperature 24 h before being provided to the larvae. Besides, feed for laying hens (PBA srl, Piacenza, Italy) was used as a control diet (cornmeal, toasted soybean meal, wheat meal, wheat bran, calcium carbonate, dicalcium phosphate, sodium chloride). Water was added to all substrates until moisture content of 50–60%. Substrates were provided to the larvae starting from a mean of 50 mg/larva/d (dry weight) and then added ad libitum avoiding competition and starvation. The mean quantity of feed (DM) provided to BSFL during the experimental period was 644 g for hen feed, 195 g for okara, 445 g for maize distillers, and 189 g for brewer’s grains. In total, 1,000 two-day-old larvae were positioned on each growing substrate, and three replications were set up. Experimental boxes (21 × 27 × 16 cm) were then closed with a perforated lid lined with fine mesh and maintained in a climate chamber (temperature 25 ± 0.5°C, relative humidity 60 ± 0.5%, light cycle 12:12 [L:D]). To study larval growth, 10 larvae from each replication of the experimental substrates were weighted separately with an analytical scale two times a week; after weighted, they were returned to their respective container. Feeding continued until 40% of the BSFL in each experimental box changed their color, indicating the reaching of the prepupal stage. When prepupae appeared, they were removed and weighted to check the final biomass produced on the different substrates.

### Substrate Reduction

In order to calculate the substrate reduction (SR), the substrate provided to the BSFL and the residue substrate were weighted. The reduction of the substrate was calculated on DM as follows: samples of the administered substrate and the residue were dehydrated in an oven at 105°C until a constant weight was reached. The SR was then calculated as in Diener et al. (2009).

### Chemical Analyses

Rearing substrates and BSFL (collected at 40% of prepupae appearance) were analyzed for the concentrations of dry matter (DM; method 945.15; AOAC International 1995), ash (method 942.05; AOAC International 1995), crude protein (CP; method 984.13; AOAC International 1995), and EE (method 920.29; AOAC International 1995). Rearing substrates were also analyzed for the concentrations of neutral detergent fiber (NDF) corrected for insoluble ash and with the addition of α-amylase (aNDFom; Mertens 2002); nonfiber carbohydrates (NFC) were calculated as 100−(ash+CP+EE+aNDFom). The BSFL were also analyzed for the concentrations of ash-free acid detergent fiber (ADF; Van Soest et al. 1991), using the Ankom 200 fiber apparatus (Ankom Technology Corp., Fairport, NY) and for N insoluble in acid detergent solution (ADIP; Licitra et al. 1996). The amount of chitin of BSFL was estimated as follows: chitin (%) = ash-free ADF (%) − ADIP (%) (Marono et al. 2015).

The experiment to assess the chemical composition and nutritive value of BSFL was replicated in three different independent experimental periods characterized by BSFL reared on the same substrates but derived from different production batch systems. At the end of each period, BSFL were freeze-dried for subsequent chemical and in vitro analysis.

### Fatty Acid Analysis

Lipid extraction from the BSFL rearing substrate and the larval meal was obtained by the cold method with methanol:chloroform 2:1 as proposed by Folch et al. (1957). The preparation of fatty acid methyl esters was performed according to Christie (2003), and samples were injected into the gas-chromatograph in split mode (split ratio 1:100). The FAME separation was performed by Thermo Trace 1300, gas chromatograph equipped by a Thermo, TR-FAME 60 m × 0.25 mm × 0.25 μm column and a flame ionization detector. The carrier gas was helium with a flow rate of 1.0 ml/min and an inlet pressure of 16.9 psi. The oven temperature program for separation was from 120 to 175°C at 10°C/min, held for 10 min and then from 175 to 230°C at 5°C/min and held for 5 min. Fatty acids were identified by comparison of retention times with standard 37 FAME mixture in dichloromethane and standard Menhaden fish oil, both obtained from Supelco (Supelco, Bellafonte, PA), and were expressed as a percentage of total fatty acids.

In vitro estimates of Dry Matter and Protein Digestibility (monogastric), and Gas Production and Organic Matter digestibility (ruminants) of BSFL

For the apparent total tract DM and CP monogastric digestibility (in vitro dDM and dCP), a three-step enzymatic method was applied to simulate the gastric, small intestine, and large intestine digestion (Boisen and Fernández 1997). At the end of the enzymatic addition, flask contents were filtered using predried (80°C) Whatman no. 54 filter papers (Whatman Inc., Florham Park, NJ) and residues dried at 80°C. The residues were weighed, and the N content was determined as previously described.

The in vitro gas production (GP) at 24 h of incubation, the OM digestibility (dOM), and Net Energy for lactation (NEl) for ruminants were calculated according to Menke and Steingass (1988) with the following equations: 

$$\begin{array}{l} dOM=9.00+0.9991\;GP+0.0595\;CP+0.0181\;ASH; \end{array}$$

$$\begin{array}{ll} NEl\;\left(MJ/kg\;of\;DM\right)= & -0.36+0.1149\;GP+0.0054\;CP\\ & +0.0139\;EE-0.0054ASH; \end{array}$$

where GP is 24 h gas net production (mL/200 mg of DM), CP, EE, and ASH are in g/kg of DM.

### Microbiome Analysis

For microbiome analysis, BSFL were fed since their hatching on the different experimental diets to avoid the influence of the hen diet on microorganism selection. The gastrointestinal tract (GIT) of mature larvae collected from each tested substrate was microdissected, washed in 70% ethanol, and immediately frozen in physiological solution at −80°C until DNA extraction. DNA from GIT was extracted using the NucleoSpin Soil kit (Macherey-Nagel). The extraction was performed with SL1 buffer and SX enhancer solutions. The extraction was done using all the GIT, i.e., without extracting the content. Each treatment was analyzed in triplicate and each extraction used three larvae. Therefore, 36 BSFL were dissected (4 substrates × 3 replications × 3 larvae). The DNA was finally recovered in 40 µl of SE buffer as suggested by the kit manufacturer.

For the identification of the bacterial community present in BSFL, a portion of the 16S gene was used, as described by Takahashi et al. (2014). For the amplification, the following primers were used: Pro341F: 5′-CCTACGGGNBGCASCAG-3′ and Pro805R: Rev 5′-GACTACNVGGGTATCTAATCC-3′. The amplifications were performed using 5 µl of the extracted DNA in a final reaction volume of 25 µl using Platinum Taq DNA polymerase high fidelity (Thermofisher). The amplifications were performed for 26 cycles using 55°C as annealing temperature.

The libraries were purified with Beads Amplure XP 0.8X, amplified with Indexes Nextera XT Illumina, normalized, mixed, and loaded on Miseq with 2 × 300 bp (paired-end) approach to generate a minimum of 50,000 sequences (±20%). The raw sequences R1 and R2 (raw reads) were verified and filtered by quality, trimmed by the primers and fused by the Qiime2 v8 software. Pipeline USEARCH and database RDP were used to obtain the taxonomic assignment.

### Statistical Analyses

Data were statistically analyzed by SAS ver 9.4. Data on larval development, total larval biomass produced, larval chemical and fatty acid composition, and nutritive value were compared by GLM procedure. Least squares mean estimates are reported. For all statistical analyses, significance was declared at *P* < 0.05 and trends at *P* < 0.10. Linear regression analysis between BSFL chemical composition and substrate composition was performed. The statistical verification of the diversity at phyla and species level between the GIT microbiome of BSFL fed with the different diets was carried out with the Kruskal–Wallis rank-sum test. If the test produces a significant *P*-value, Dunn tests were conducted to discern which of many possible sample pair combinations were significantly different with a *P* < 0.05.

## Results

### Chemical Composition of the Rearing Substrates

The average chemical composition of the rearing substrates is reported in Table 1. All the substrates were characterized by wide variability in terms of chemical composition for all the analyzed parameters. The diets were characterized by a significant amount of CP (from 15.8 of brewer’s grains to 39.2% on DM for okara). Hen diet, as expected, was characterized by a high level of ash (13.5% on DM). Okara diet had the highest EE content (17.2% on DM), followed by maize distillers (11.1%), hen (4.00%), and brewer’s grains (2.89). Fiber content (% on DM) ranged from 15.7 (hen diet) to 53.6 (brewer’s grains). The NFC content (% on DM) ranged from 7.47 (okara) to 49.8 (hen diet) with brewer’s grains and maize distillers being intermediate.

**Table 1.** Chemical composition (% DM unless otherwise stated) and fatty acid composition (% total fatty acids) of rearing substrates

	Hen diet	Okara	Maize distillers	Brewer’s grains
DM (%)	92.1	18.3	94.9	15.8
Ash	13.5	4.13	5.40	4.13
CP	17.0	39.2	29.5	15.8
EE	4.00	17.2	11.1	2.89
NDF	15.7	32.0	36.7	53.6
NFC	49.8	7.47	17.3	11.2
C12:0	0.00	0.12	0.00	0.09
C14:0	0.10	0.10	0.00	0.32
C16:0	18.7	10.8	14.4	25.9
C16:1 n-7	0.25	0.12	0.13	0.00
C18:0	2.79	4.87	2.04	2.02
C18:1 n-9 cis	29.1	27.7	26.6	10.6
C18:1 n-7	1.10	1.41	0.68	0.72
C18:2 n-6 cis 9,12	43.7	48.0	53.9	53.5
C18:3 n-3	2.47	5.84	1.59	5.68

The main fatty acids were: palmitic (16:0), linoleic (18:2 n-6 cis 9,12), and oleic (18:1 n-9 cis). Palmitic acid had the highest concentration among saturated fatty acids with differences among substrates: 10.8-14.4-18.7 and 25.9% of total fatty acid for okara, maize distillers, hen diet, and brewer’s grains, respectively. Linoleic acid was the fatty acid with the highest concentration (from 43.7 for hen diet to 53.9 % of total fatty acid for maize distillers) followed by oleic (from 10.6 for brewer’s grains to 29.1 for hen diet) and linolenic (from 2.47 for hen diet to 5.84 for okara).

### BSF Growth, Biomass Production, and SR

**Fig. 1.**
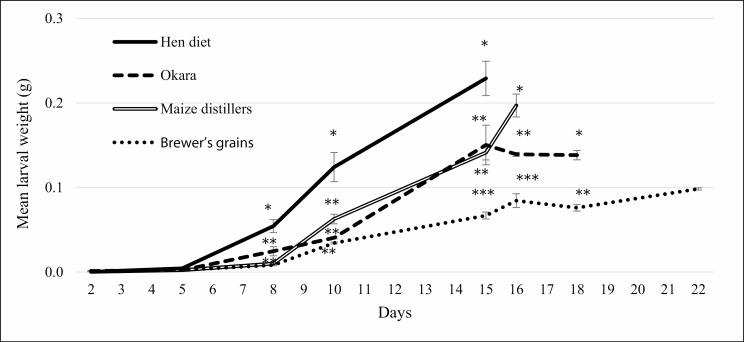
Mean BSF larval weight (g ± SE) on the experimental substrates. Different number of asterisks indicates significant differences among larval weights at each measurement point (*P* < 0.05).

All the tested agro-industrial byproducts were suitable for the growth and development of BSFL. We analyzed the trend of larval growth until BSFL reached prepupal instar (Fig. 1). The BSFL reared on the hen diet gained weight starting from the fifth days from the beginning of the trial, while on the other byproducts, the growth was slower, particularly on the brewer’s grains, as illustrated from the slope of the growth curve. Significant differences in larval weight were observed starting from day 8, with the BSFL on hen diet heavier than the others. After 15 d, BSFL on brewer’s grains were significantly lighter than larvae on the other substrates, and this trend was confirmed up to the end of the trial. Time to reach 40% of prepupal instar was shorter on the control diet, followed by maize distillers and okara; on brewer’s grains the development time was the longest, up to 22 d, and this byproduct was statistically different from the others (*P* < 0.05).

No statistical differences were noticed among larval survival on the different growing substrates, albeit higher mortality was registered on maize distillers compared with the other diets (Table 2). Larval biomass yielded at the end of the experiment was higher on the hen diet and statistically similar to that collected on maize distillers. Larvae grown on these two substrates showed the maximum weight compared with the others. Only 82.61 and 79.63 g of fresh biomass were collected on okara and brewer’s grains, respectively.

**Table 2.** Larval survival (%), biomass yield (g) (WM and DM), and SR (g DM/1,000 larvae) of BSFL reared on experimental substrates

	Hen diet	Okara	Maize distillers	Brewer’s grains	SEM	*P*	*F* (dF_3, 11_)
Larval survival (%)^*a*^	97.5	98.5	73	95.9	4.03	>0.005	4.01
Biomass yield (g) (WM)	169a	82.6b	144a	79.6b	15.1	0.007	8.74
Biomass yield (g) (DM)	65.6a	31.2bc	55.5ab	28.3c	5.4	0.005	9.80
SR (g DM/1000 larvae)	0.58	0.73	0.45	0.60	0.03		

Different letters in the same row indicate a difference for *P* < 0.05.

^*a*^Data from Bava et al. (2019).

The SR was different within the different tested diets. A maximum of 0.73 g (DM)/1,000 larvae of SR was assessed on okara, whereas on maize distillers it was only 0.45 g (DM)/1,000 larvae. Intermediate values were observed on hen feed and brewer’s grains, with a mean of 0.58 and 0.60g (DM)/1,000 larvae, respectively.

### Chemical Composition of BSFL Reared on the Experimental Substrates

Larvae chemical and fatty acid compositions are reported in Table 3 and refer to BSFL collected at 40% of prepupae appearance. Larvae were characterized by high CP content (50.6% on DM on average), which was not affected by the rearing substrate (*P* = 0.470). The ash content was affected by the rearing substrate (*P* = 0.008) with the highest value (13.5% on DM) for BSFL grown on hen diet in comparison with the other treatments (on average, 6.04% on DM). The variability for ash content of BSFL expressed as coefficient of variation among treatments was very high and equal to 43.1%. Similarly, the EE concentration of BSFL was affected by the rearing substrate (*P* = 0.040). Particularly, EE content (% DM) was higher for BSFL grown on okara (32.1) although not different (*P* > 0.05) than BSFL grown on maize distillers (30.3). The BSFL reared on brewer’s grains had a EE content (25.1) lower than that grown on okara and maize distillers. The EE content of hen BSFL (26.0) was statistically lower than okara BSFL (*P* < 0.05). The variability for EE content of BSFL expressed as coefficient of variation among treatments was equal to 16.2%.

**Table 3.** Chemical composition (% DM unless otherwise stated) and fatty acid composition (% total fatty acids) of BSFL reared on different substrates

	Hen diet	Okara	Maize distillers	Brewer’s grains	SE	*P*	*F* (dF_3, 11_)
DM (%)	38.9	37.4	38.4	35.6	0.846	0.119	2.98
Ash	13.5a	5.83b	4.62b	7.68b	1.21	0.008	10.7
CP	48.2	49.8	51.7	52.5	1.95	0.470	0.97
EE	26.0bc	32.1a	30.3ab	25.1c	1.51	0.040	4.99
Chitin^*a*^	3.73	3.39	4.40	6.34	0.62	0.057	4.47
C12:0	37.9a	17.6b	31.6a	28.9ab	4.28	0.035	6.57
C14:0	7.94a	3.34b	6.61a	5.83ab	0.86	0.021	8.36
C16:0	16.7	14.0	13.6	20.5	2.07	0.146	2.83
C16:1 n-7	2.63	1.54	1.1	4.26	0.71	0.064	4.73
C18:0	2.77	3.26	2.1	3.10	0.41	0.182	2.42
C18:1 n-7 cis	0.66bc	0.91ab	0.24c	1.06a	0.17	0.033	6.77
C18:1 n-9 cis	11.6b	23.5a	15.9b	11.2b	2.06	0.011	11.7
C18:2 n-6 cis 9,12	14.7	28.4	24.3	21.4	3.78	0.090	3.87
C18:3 n-3	1.15	2.85	0.72	1.92	0.55	0.065	4.69
C20:0	1.55	2.58	2.17	0.017	0.71	0.138	2.94
Total saturated	68.3a	41.3c	56.9b	60ab	4.12	0.009	12.4

Different letters in the same row indicate a difference for *P* < 0.05.

^*a*^Estimated chitin, according to Marono et al. (2015).

The relationship between BSFL EE concentrations and that of the substrate was as follows:

$$\begin{array}{ll} BSFL\;EE\;concentration\;(\%\;DM)=\; & 0.497\;Diet\;EE(\%\;DM)\\ & +24.0\;(r^{2}=0.973\;RMSE=0.681;\;\\ & P=0.014,\;n=12) \end{array}$$

A tendency (*P* = 0.057) for a higher chitin concentration (% on DM) of brewer’s grains BSFL (6.34) in comparison with hen (3.73), okara (3.39), and maize distillers BSFL (4.40) was observed. The variability for chitin content of BSFL expressed as coefficient of variation among treatments was equal to 25.6%. The results of the present study underlined a positive relationship between larvae chitin content and NDF content of rearing substrate as follows:

$$\begin{array}{ll} BSFL\;chitin\;(\%\;DM)= & \;0.0715\;Diet\;NDF(\%\;DM)\\ & +1.997\;(r^{2}=0.716;\;RMSE=0.862;\;\\ & P=0.15;\;n=12). \end{array}$$

Total saturated fatty acid content of BSFL was affected by rearing substrate (*P* = 0.009) with the highest value for hen BSFL (68.3%), although not different than brewer’s grains BSFL (60.0%). The maize distillers BSFL content of saturated fatty acids (56.9%) was similar to brewer’s grains BSFL and the lowest value (41.3%) was obtained for okara BSFL. Among the saturated fatty acids, lauric acid (C12:0) was present with the highest concentration, but there was a difference depending on the rearing substrate (*P* = 0.035). Particularly, hen BSFL were characterized by a higher value (37.9% of total fatty acid) than okara BSFL (17.6). Among saturated fatty acid, myristic acid (C14:0) was also affected by rearing substrate (*P* = 0.021), whereas palmitic acid (C 16:0) and stearic acid (C18:0) were not affected by rearing substrate. Among the unsaturated fatty acids, linoleic acid (C18:2) was present in a higher amount, with a tendency (*P* = 0.09) for lower concentration in hen BSFL (14.7% total fatty acids) than other BSFL (on average 24.7% total fatty acids). Oleic acid (C18:1 n-9) was also determined in high concentrations (>10% for all BSFL) with a difference depending on the rearing substrate (*P* = 0.011) with the highest value for okara BSFL (23.5% total fatty acids) than other BSFL (on average 12.9% total fatty acids). A difference was also observed for vaccenic acid (C18:1 n-7) (*P* = 0.033) with a higher value for brewer’s grains BSFL (1.06% total fatty acids) as compared with hen and maize distillers BSFL (0.66 and 0.24% total fatty acids, respectively).

### In vitro Estimates of Digestibility for Monogastrics and Ruminants

The results of in vitro digestibility for monogastric are in Table 4. The dDM (%) of larvae was affected by rearing substrate (*P* = 0.039), with higher values for okara and maize distillers (85.2) as compared with hen BSFL (80.2); brewer’s grains BSFL (82.2) were intermediate and not different from the other treatments. Similarly, there was an effect of rearing substrate for the dCP of BSFL (%; *P* = 0.010), which was highest for BSFL reared on maize distillers (87.8), intermediate for brewer’s grains (85.5), and okara (85.7) BSFL, and the lowest for hen BSFL (82.7).

**Table 4.** Nutritive value for monogastrics and ruminants of BSFL reread on different diets

	Hen diet	Okara	Maize distillers	Brewer’s grains	SE	*P*	*F* (dF_3, 11_)
In vitro dDM	80.2b	85.2a	85.2a	82.2ab	1.07	0.039	5.35
In vitro dCP	82.7c	85.7ab	87.8a	85.5b	0.67	0.010	9.62
Gas production 24 h	21.8	21.5	20.3	22.8	0.86	0.265	1.79
dOM^*a*^	61.9	61.1	60.9	64.6	1.15	0.179	2.45
Net energy (MJ/kg DM)^*a*^	7.624b	8.941a	8.737a	8.204ab	0.243	0.016	9.71

^*a*^Calculated from gas production according to Menke and Steingass (1988).

Different letters in the same row indicate a difference for *P* < 0.05.

The results of the present study underlined a negative relationship between larvae dCP and ash content of rearing substrate as follows:

$$\begin{array}{ll} BSFL\;dCP(\%)=\; & -0.4268*Diet\;Ash\;(\%\;DM)\\ & +88.8\;(r^{2}=0.687;\;RMSE=1.305;\;\\ & \;P=0.002;\;n=12). \end{array}$$

Considering the nutritive value for ruminants, the only difference (*P* = 0.016) was observed for Net Energy for lactation (MJ/kg DM) with a lower value for hen BSFL (7.62) as compared with okara (8.94) and maize distillers BSFL (8.74). The brewer’s grains BSFL had an intermediate value (8.20).

### Microbiome Analysis

In total, 262,395 filtered sequences were obtained after sequencing, with an average of 21.866 reads/sample. Referring GIT microbiota composition in terms of phyla, the larvae developed a diversified microbiota. The statistical analysis of the phyla content differences is indicated in Table 5 (Kruskal–Wallis rank-sum test). The bacteria belonging to the Proteobacteria phylum represents the majority of the microbiota colonizing GIT. The BSFL reared on okara represented an exception since, for this treatment, most of the bacteria belong to the phylum of Bacteroidetes. Bacteroidetes were present in appreciable percentages in the GIT of BSFL reared with three diets (okara, brewers’s grains, and hen), whereas they were absent in the GIT of the BSFL reared with maize distillers. Firmicutes had a similar characteristic, being present in three diets but almost absent in the GIT of the BSFL bred with brewer’s grains. Finally, also Actinobacteria were present in a very low percentage in the larvae fed on the brewer’s grains, even if, in this case, the observed differences were not statistically different.

**Table 5.** Comparison of phylum composition in the GIT of BSFL fed with the four substrates (% of the total number of bacteria present, as mean ± SE is reported)

	Hen diet	Okara	Maize distillers	Brewer’s grains	*P*	*F* (dF_3, 11_)
Proteobacteria	58.5 ± 2.4ab	38.3 ± 1.7b	84.1 ± 8.5a	77.1 ± 8.2ab	0.021	9.67
Firmicutes	20.3 ± 4.6a	6.6 ± 5.6ab	9.5 ± 5.9ab	0.5 ± 0.3b	0.027	7.21
Bacteroidetes	8.5 ± 6.0ab	52.6 ± 3.2b	0.0 ± 0.0a	21.5 ± 9.3ab	0.036	8.56
Actinobacteria	12.6 ± 3.6	2.5 ± 1.3	6.3 ± 5.0	0.8 ± 0.9	0.16s	5.05
Not identified	0.1 ± 0.1	0.0 ± 0.0	0.0 ± 0.1	0.0 ± 0.0		

Different letters in the same row indicate a difference for *P* < 0.05.

Regarding the microbial complexity, the greatest value was observed in the GIT of the BSFL bred with the hen diet. Considering only the species present in a percentage greater than 0.5%, the larvae grown on the hen diet showed an average of 15.7 species while the larvae fed with the other diets showed lower values (brewer’s grains = 10.6; maize distillers = 9.7, and okara = 10.3).

In total, 24 species of bacteria were identified, and only *Providencia vermicola*, *Morganella morganii* subsp. *morganii*, and *Klesbiella pneumoniae* subsp*. ozaenae* were present in the GIT of all larvae grown on the four substrates (Table 6). *Lactobacillus dextrinicus* (4.4%), *Rhodobacter maris* (6.1%), *Corynebacterium aurimucosum* (4.3%), and *Sphingobacterium lactis* (7.9%) were found only in the BSFL GIT of hen diet. *Orbus sasakiae* (3.2%) was only found in BSFL GIT reared on okara, whereas *Clostridium amygdalinum* was found on okara samples (5.7%) and in traces in brewer’s grains (0.1%). The presence of Serratia proteamaculans and Sphingobacterium cladoniae was significantly higher in BSFL GIT grown on brewer’s grain than on other diets. Finally, only one bacterial species was present in all diets other than the control: *Campylobacter coli* (Table 6).

**Table 6.** Bacteria species identified in the GIT of BSFL larvae fed with the four substrates (% of the total number of bacteria present as mean ± SE)

Species	Hen diet	Okara	Maize distillers	Brewer’s grains	*P*	*F* (dF_3, 11_)
*Campylobacter coli*	0.0 ± 0.0a	1.8 ± 1.6ab	73.3 ± 11.6b	24.6 ± 14.2ab	0.021	9.67
*Dysgonomonas capnocytophagoides*	0.3 ± 0.3ab	27.3 ± 12.4b	0.0 ± 0.0a	17.4 ± 9.4b	0.025	9.36
*Dysgonomonas macrotermitis*	0.1 ± 0.0ab	25.2 ± 12.7b	0.0 ± 0.0a	0.5 ± 0.3ab	0.015	10.53
*Providencia vermicola*	1.1 ± 0.2a	13.1 ± 5.0b	1.2 ± 0.8a	9.1 ± 1.3ab	0.038	8.44
*Morganella morganii* subsp*. morganii*	3.4 ± 2.2	16.5 ± 1.9	1.4 ± 0.9	5.2 ± 3.4	0.319	3.51
*Klebsiella pneumoniae* subsp*.ozaenae*	41.6 ± 3.1a	1.3 ± 0.5b	6.6 ± 3.1ab	28.1 ± 3.1ab	0.033	8.74
*Weissella paramesenteroides*	7.3 ± 0.6a	0.0 ± 0.0b	1.6 ± 0.8ab	0.0 ± 0.0b	0.014	10.65
*Clostridium amygdalinum*	0.0 ± 0.0a	5.7 ± 6.0b	0.0 ± 0.0a	0.1 ± 0.1ab	0.028	9.07
*Corynebacterium nuruki*	7.8 ± 1.7a	0.0 ± 0.0b	2.3 ± 1.2ab	0.0 ± 0.0b	0.021	9.70
*Enterococcus saccharolyticus*	4.1 ± 1.5ab	0.6 ± 0.8ab	6.3 ± 4.5a	0.3 ± 0.3b	0.033	8.74
*Lactobacillus dextrinicus*	4.4 ± 2.5a	0.0 ± 0.0b	0.0 ± 0.0b	0.0 ± 0.0b	0.017	10.17
*Rhodobacter maris*	6.1 ± 0.6a	0.0 ± 0.0b	0.0 ± 0.0b	0.0 ± 0.0b	0.014	10.65
*Corynebacterium aurimucosum*	4.3 ± 2.4a	0.0 ± 0.0b	0.0 ± 0.0b	0.0 ± 0.0b	0.014	10.73
*Sphingobacterium lactis*	7.9 ± 6.2a	0.0 ± 0.0b	0.0 ± 0.0b	0.2 ± 0.0b	0.015	10.53
*Actinomyces odontolyticus*	0.4 ± 0.2	1.3 ± 1.2	3.7 ± 3.3	0.2 ± 0.3	0.176	4.95
*Orbus sasakiae*	0.0 ± 0.0a	3.2 ± 1.7b	0.0 ± 0.0a	0.0 ± 0.0a	0.025	9.31
*Serratia proteamaculans*	0.2 ± 0.1a	0.0 ± 0.0a	0.0 ± 0.0a	4.9 ± 1.2b	0.015	10.53
*Enterococcus diestrammenae*	2.4 ± 0.9a	0.2 ± 0.2b	0.2 ± 0.2b	0.0 ± 0.0b	0.041	8.23
*Actinomyces marimammalium*	0.1 ± 0.1	1.2 ± 1.0	0.4 ± 0.7	0.6 ± 1.0	0.319	3.51
*Proteus mirabilis*	2.7 ± 0.2	0.0 ± 0.0	0.7 ± 1.3	0.3 ± 0.2	0.055	7.62
*Sphingobacterium cladoniae*	0.2 ± 0.1a	0.0 ± 0.0a	0.0 ± 0.0a	3.5 ± 0.3b	0.014	10.57
*Salmonella enterica* subsp*. arizonae*	0.9 ± 0.2ab	2.4 ± 1.5ab	0.5 ± 0.2b	3.1 ± 1.4a	0.022	9.67
*Raoultella planticola*	2.6 ± 0.2a	0.0 ± 0.0b	0.4 ± 0.3ab	1.8 ± 0.6ab	0.017	10.12
*Enterococcus lactis*	2.2 ± 0.8a	0.0 ± 0.0b	1.4 ± 0.8ab	0.0 ± 0.0b	0.033	8.74

Different letters in the same row indicate a difference for *P* < 0.05.

As the last aspect, a possible correlation between components of the diet and microbial communities was evidenced. The development of a bacterial community is essentially based on a competition between different species. Therefore, it is possible that the presence of a specific component in the diet can favor a phylum, which will tend to be present in a greater percentage. We analyzed the regression between ashes, CP, ether extract, NDF, and NCF versus the phyla present in the GIT. Considering four observations, the only value to check statistical significance is *P* < 0.1 if *r* > 0.8 (Spearman’s rank correlation coefficient for a small sample). Ashes correlate positively with both Firmicutes and Actinobacteria (*r* = 0.93 and 0.94, respectively). NDF correlates negatively with both Firmicutes and Actinobacteria (*r* = −0.95 and −0.87, respectively), whereas the same phylum also correlates, but positively, with NCF (*r* = 0.91 and 0.95).

## Discussion

### Larvae Growth Performance

The present study aimed to evaluate the effect of the rearing substrate on BSFL biomass production, nutritive value, and gut microbiome. The assessment of the nutritional requirements of BSFL and the evaluation of alternative agro-industrial byproducts to obtain optimal BSFL performance in terms of development, waste reduction efficiency, and nutritional composition are fundamental for a sustainable mass-rearing system (Spranghers et al. 2017, Meneguz et al. 2018, Chia et al. 2020). The use of agro-industrial byproducts for animal feeding can be limited due to constraints such as variation in nutrient composition and technical requirements for preservation (Salami et al. 2019). However, these constraints seem less critical for insect rearing, particularly for a saprophagous species as BSF, although this should be evaluated.

The agro-industrial byproducts tested in the present study were all suitable for BSFL rearing and characterized by wide variability in chemical composition (CP, EE, and NDF), thus influencing performance, composition, and nutritive value of the larvae. Larvae showed a high survival rate on all the experimental substrates, and the heaviest larvae were observed on the hen diet and maize distillers (Bava et al. 2019). The larval developmental time recorded on okara was in line with data reported by other authors (Lim et al. 2019), whereas on brewer’s grains, Chia et al. (2020) reported a lower number of days necessary to reach the prepupal stage. The longest time required for larval growth on brewer’s grains of the present experiment is probably due to the high fiber content, relatively hard to digest, as already observed by ur Rehman et al. (2017). As far as we know, no other data on larval development on maize distillers are available. Considering the capability of larvae to reduce the given substrate, our results are in line with results observed on vegetable substrates as sugar beet pulp, fermented maize straw, fruits, and vegetables (Tschirner and Simon 2015, Gao et al. 2019, Lalander et al. 2019). This ability suggests the possibility of using BSFL for the valorization and the management of the byproducts considered in this study.

### Larvae Chemical Composition and Nutritive Value

The results confirmed that the rearing substrate affected both the chemical composition and nutritive value of BSFL. The chemical parameters mostly affected by the rearing substrate were the ash and EE contents. Moreover, chitin seemed to be positively correlated with the NDF content of the substrate. To the best of our knowledge, no trials studied the relationship between diet composition and BSFL chitin content. The BSFL can partially digest NDF, but fiber utilization efficiency is lower than that of nonfibrous carbohydrates resulting in a slower growth and a smaller final body mass for BSFL grown on brewers’s grains (higher NDF content) in comparison with the other treatments. Consequently, there was a higher surface: volume ratio for BSFL grown on brewer’s grains compared with the other treatments, which possibly increased the proportion of exoskeleton chitin in brewer’s grains BSFL.

Excessive chitin content could decrease BSFL CP digestibility. However, recent findings (Tabata et al. 2018) show that omnivores possess high chitin digestion ability in their gut compared with carnivores and herbivores. Moreover, as recently reviewed by Swiatkiewicz et al. (2015), chitosan (originated by deacetylated chitin) has beneficial effects such as immunomodulatory, antioxidative, and antimicrobial properties, which in many studies were related to the improved growth performance of piglets and poultry (Swiatkiewicz et al. 2015). As concluded by Shi et al. (2005), in poultry species, there is a threshold inclusion (5 g/kg), where chitosan supplementation changes from being beneficial to detrimental in poultry diets, decreasing N utilization efficiency.

The mean estimated chitin content of BSFL in the present study agrees with Finke (2013) (5.41% on DM), and this value is slightly lower than the average value (6.02% DM) reported by Spranghers et al. (2017).

The CP content of BSFL was not affected by the substrate. The CP values were computed from N using a conversion factor equal to 6.25. The presence of Non-Protein Nitrogen in insects (e.g., nucleic acids, phospholipids, and excretion products in the intestinal tract) could lead to an overestimation of the actual protein content (Mariotti et al. 2008). For this reason, recently, Janssen et al. (2017) determined a conversion factor of 4.76 to quantify protein content in whole larvae. Applying this conversion factor, the CP content (% DM) of BSFL would be 36.7 (hen diet), 37.9 (okara), 39.4 (maize distillers), and 40.0 (brewer’s grains).

Despite a similar CP content, protein digestibility was affected by BSFL ash concentration. The dCP values were slightly lower than the value reported by Bosch et al. (2016) and Ottoboni et al. (2017), but higher than the values reported by Marono et al. (2015). The difference in dCP depending on the rearing substrate could be due to different mineral content in chitin. The cuticle of insects consists of alternate protein layers and chitin impregnated with calcium carbonate (Whistler 1993). Notably, in the present study, the hen diet was characterized by high ash and calcium (4.32% on DM) contents, which could have increased the calcium carbonate content of chitin, resulting in a lower dCP for BSFL reared on hen diet. We hypothesize that calcium carbonate can protect the protein-chitin, which overall is less digested. With this regard, the conventional extraction of chitin is performed chemically by demineralizing the exoskeleton shells by means of strong acids and subsequently removing residual protein through incubation with a strong base. Based on our results, it can be hypothesized that the in vitro dCP is not affected by total chitin content but probably by the mineral/calcium content of chitin itself. Hence, since rearing substrate ash content strongly affects that of BSFL, using diets with low ash content is advisable also in order to improve protein digestibility.

Considering the nutritive value for ruminants, unlike monogastrics, the in vitro rumen dOM was not affected by the rearing substrate, and the obtained values were lower than the in vitro DM true digestibility for ruminants obtained by Campbell et al. (2020), but higher than the values for OM reported by Jayanegara et al. (2017).

In the literature, not many studies determined the nutritive value of BSFL for ruminants. The results of our study aimed to provide a preliminary characterization of BSFL nutritive value based on GP method. Overall, the difference in NEl content observed was due to the different chemical composition of BSFL, rather than to a different fermentability in the rumen, as confirmed by GP data.

Similarities between the fatty acid composition of BSFL and that of the substrate were recorded for palmitic, oleic, and linoleic fatty acids, some of the most prevalent fatty acids found in the substrates. Despite the rearing substrates being rich in palmitic and oleic acid, a recent study (Hoc et al. 2020) demonstrated that BSFL can partially produce these fatty acids via biosynthesis pathways and not only by diet accumulation. On the other hand, other fatty acids, such as lauric or myristic fatty acids, were present in BSFL but not in the rearing substrates. Lauric acid was the fatty acid present with the highest concentration, in agreement with other studies (Spranghers et al. 2017, Campbell et al. 2020), and it was synthesized from other nutrients present in the substrate, such as carbohydrates (starch and sugars) (Spranghers et al. 2017, Ewald et al. 2020). Moreover, recent evidence (Hoc et al. 2020) suggested the importance of carbohydrates as a source of acetyl-CoA in the BSFL fatty acid profile constitutions; the authors underlined the need to conduct further studies on the role of carbohydrate level in BSF diets as they are an essential source of acetyl-CoA, a critical molecule in the biosynthesis of fatty acids. In accordance with the biochemical pattern illustrated by Hoc et al. (2020), BSFL grown on the hen diet, highest in NFC and hence with a probably higher acetyl-CoA production, contained the highest levels of lauric acid (37.9%), whereas BSFL grown on the okara (only 7.47 % NFC) contained the lowest lauric acid level and the highest levels of oleic and linoleic fatty acids. Referring again to the biochemical pathways described by Hoc et al. (2020), the higher acetyl-CoA associated with a high carbohydrate content may be the reason for the higher saturated fatty acid content of BSFL on the hen diet.

Despite being a saturated fatty acid, hence undesirable, lauric acid is known for being particularly active against Gram-positive bacteria (Dierick et al. 2002). Therefore, the possible antimicrobial effects of BSFL fat could provide a significant added value when whole larvae/prepupae are used as a protein source in the feed of monogastrics, especially in the early phase (Spranghers et al. 2017, 2018).

### Microbiota Composition

As concern BSFL microbiota, it is evident that the diet administered to the larvae radically changed the composition of the microbiota, confirming what was previously observed (Jeon et al. 2011, Bruno et al. 2019). Compared with other studies, the abundant presence of Proteobacteria and Firmicutes was here confirmed, whereas no bacterium belonging to the phylum of the Tenericutes and Actinobacteria was found in our study.

Related to the species presence, only three were present in the GIT of the larvae grown on the four substrates: *Providencia vermicola*, *Morganella morganii* subsp. *morganii*, and *Klesbiella pneumoniae* subsp*. ozaenae*. *Klesbiella pneumoniae* was identified as one of the most present species in the GIT of BSFL grown on cooked rice (Jeon et al. 2011), whereas in the same report, *M. morganii* was identified as abundant in larvae fed with calf forage. Callegari et al. (2020) documented the presence of several strains belonging to the genus *Providencia* in BSFL reared on a fruit diet (apple, pear, and orange).

The greater complexity of the hen diet compared with other tested substrates is also attested by the presence in the BSFL GIT of several species not found in the other analyzed samples. Our results agree with what other authors have observed: when diets were very unbalanced, the diversity of microbial communities decreased compared with those in nutritionally more balanced diets (Jeon et al. 2011, Bruno et al. 2019). We verified that the control diet, i.e., hen diet, induced the greatest complexity in BFL intestinal microbiota. Finally, the presence of the different phylum was correlated with the diet composition: protein and lipid concentration of diets did not seem to influence the development of a particular phylum, whereas carbohydrates (both NDF and NCF) had a great influence.

In conclusion, the present study confirms that the growing substrate influences insect nutritional profile for several parameters such as lipid and ash concentration, in vitro digestibility, saturated fatty acid content, and estimated chitin. In this view, dietary manipulation can contribute to an insect meal production with high and constant quality, overcoming some negative issues related to insect quality, such as a high saturated fatty acid content. With this regard, the okara byproduct seems to be the most interesting, supporting both BSFL growth and low saturated fatty acid content of BSFL.

In addition to their nutritional and dietetics features, BSFL also represents a way to convert agro-industrial byproducts into valuable feed materials sustaining the circular economy. Due to the new knowledge gained about chitinase activity in the monogastric gut, possible modification of the in vitro methods, such as the inclusion of chitinase, can be foreseen.
